# Drug sensitivity in cancer cell lines is not tissue-specific

**DOI:** 10.1186/s12943-015-0312-6

**Published:** 2015-02-15

**Authors:** Samira Jaeger, Miquel Duran-Frigola, Patrick Aloy

**Affiliations:** Joint IRB-BSC-CRG Program in Computational Biology, Institute for Research in Biomedicine (IRB Barcelona), c/Baldiri i Reixac 10-12, Barcelona, 08028 Catalonia Spain; Institució Catalana de Recerca i Estudis Avançats (ICREA), Pg. Lluís Companys 23, Barcelona, 08010 Catalonia Spain

**Keywords:** Cancer cell lines, Tissue specificity, Drug sensitivity

## Abstract

**Background:**

Cancer cell lines have a prominent role in the initial stages of drug discovery, facilitating high-throughput screening of potential drugs. However, their clinical relevance remains controversial.

**Findings:**

We assess whether drug sensitivity in cancer cell lines is able to discriminate tissue specificity. We find that cancer-specific drugs do not show higher efficacies in cell lines representing the respective tissues. Even when considering distinct cancer subtypes and targeted therapies, most drugs are evenly effective/ineffective throughout all cell lines.

**Conclusions:**

To get the most out of cell line panels, it will be necessary to look into their molecular characteristics, and integrate them into systems biology frameworks.

**Electronic supplementary material:**

The online version of this article (doi:10.1186/s12943-015-0312-6) contains supplementary material, which is available to authorized users.

## Findings

Human cancer cell lines are widely used *in vitro* models for studying cancer and its biology [1]. Apart from being valuable tools for identifying biomarkers and genetic variants that impact drug sensitivities [2,3], cancer cell lines play a pivotal role in the early stages of drug discovery, facilitating the screening of hundreds of potential drugs and their combinations before translating the outcomes into *in vivo* models and expensive clinical trials [4,5].

**Figure 1:**
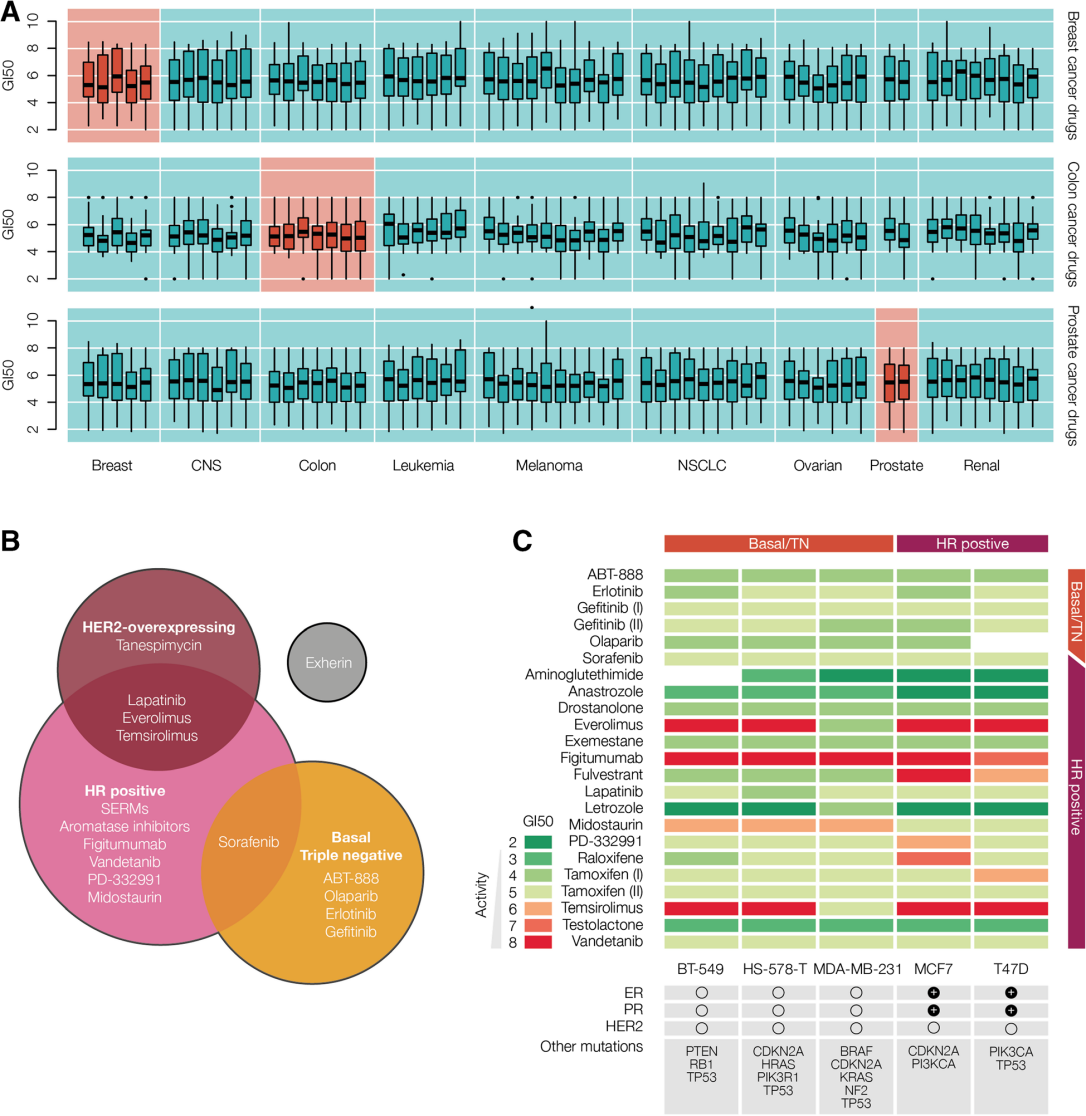
**Tissue specificity displayed by caner-specific drugs. (A)** Sensitivity of cancer-specific drugs intended for breast cancer (41), colorectal cancer (24) and prostate cancer (38) across the NCI-60 panel covering 59 cancer cell lines derived from nine cancer tissues. The GI50 represents the negative log of the concentration that is required to inhibit the growth of a cell line by 50%. Each box represents the distribution of drug activities in a given cell line. Cell lines are organized in tissue slots. In red, we highlight the cell lines representing the intended tissue of the drugs. **(B)** Breast cancer subtype-specific analysis. Stratification of targeted agents according to the different breast cancer subtypes. Note that agents might be used for more than one subtype. **(C)** Sensitivity of HR-positive and triple negative breast cancer drugs with respect to subtype-specific breast cancer cell lines. Red indicates high drug activity while green indicates inactivity for a given cell line.

However, despite their fundamental role in biomedical research, the clinical relevance of cell lines remains highly controversial [6,7]. Apart from known technical and biological limitations, like contamination, missing tumor microenvironment, or lack of drug distribution and metabolism, the main concern is whether cancer cell lines are true representatives of primary tumors [8]. Prolonged culturing of immortalized cell lines may induce extensive modifications in their molecular characteristics, like secondary genomic changes [7,9], and it remains unclear how closely they still resemble the original tissue after undergoing a certain number of passages. To complement the ongoing debate from a pharmacological perspective, we analyzed the predictive power of cancer cell lines for elucidating cancer-specific drug responses. Considering the widely used NCI-60 panel [5], we assessed whether drugs for a particular cancer type display a higher efficacy in cell lines supposedly representing the respective cancer tissue. We focused on three cancer types, namely, breast, colorectal and prostate cancer, represented by five, seven and two cell lines, respectively, in the NCI-60. We analyzed all the 75 compounds, approved or experimental, associated with the treatment of these cancers that have also been tested in the NCI-60 (Additional file 1: Table S1 and Additional file 2: Table S2). We examined the sensitivity of each drug by considering its GI50 across the complete NCI-60 panel, derived from nine cancer tissues. The GI50, similar to IC50 and EC50, indicates the concentration required to inhibit cell proliferation by 50%, relative to untreated samples [5]. Note that we studied the activity of final drugs instead of the preliminary hit compounds expecting that the former remain active after preclinical optimization. Figure 1A shows the sensitivity of the corresponding cancer cell lines towards cancer-specific agents (highlighted in red) in comparison to the sensitivity measured in cell lines representing other cancer tissues. Surprisingly, cancer-specific drugs do not show a significantly higher activity in cell lines representing the respective tissue (Wilcoxon test with Bonferroni correction for multiple testing; see Additional file 1). The same trend can be observed when focusing only on approved drugs or more specific targeted agents, and after normalizing drug sensitivity values across cell lines. To avoid biases caused by drugs with multiple therapeutic indications, we also analyzed more restrictive sets containing compounds exclusively associated with breast, colorectal or prostate cancers, obtaining equivalent outcomes (Additional file 1: Figures S1, S2 and S3). Overall, our results indicate that a simple selection of cell lines according to the tissue of interest does not reflect the eventual complexity in the clinics.

Given the intrinsic heterogeneity of most cancers [10], no individual cell line is likely to be a general representative for the distinct cancers derived from one tissue [11]. Hence, we do not expect a tissue-specific drug to perform equally well across the corresponding cell lines. Breast cancer, for instance, is a heterogeneous disease with at least four recognized subtypes that require a specific treatment [12,13]. Most researchers select particular cell lines based on receptor status, common genetic mutations, molecular signatures, tumor type, as well as technical or methodological limitations for their experiments [14]. Thus, mimicking this common approach, we considered the presence or absence of biomarkers that define the distinct breast cancer subtypes. Next we examined whether subtype-specific responses are reflected in cell lines representing distinct subtypes. The NCI-60 covers two subtypes, the triple-negative breast cancer (BT-549, HS-578 T and MDA-MB-231) and the HR-positive breast cancer subtype (MCF7 and T-47D). Neither the HER2-overexpressing nor the normal-like subtypes are included in the panel. Among the 25 targeted breast-cancer agents, 18 are indicated for HR-positive patients, 6 for triple-negative and 4 for HER2-overexpressing breast cancer (Figure 1B and Additional file 1: Table S3). According to this stratification, we assessed the sensitivity of the subtype-specific drugs in the corresponding cell lines. Figure 1C shows that the majority of drugs is equally active or inactive independent of the breast cancer subtype. Figitumumab, vandetanib, and gefitinib (I), for example, are evenly effective in HR-positive and triple-negative cell lines, while aromatase inhibitors like letrozole, anastrozole and aminoglutethimide are basically inactive and would not have been discovered through screening cell lines from this panel. Only four out of 18 HR-positive drugs, i.e., PD-332991, raloxifene, tamoxifen (I), and fulvestrant, exhibit a higher specificity in at least one of the HR-positive cell lines. Conversely, midostaurin, a targeted drug against HR-positive tumors, only shows activity in triple-negative cell lines. As the NCI-60 only involves five breast cancer cell lines, which only partially reflect the distinct subtypes, we performed the same analysis on the more comprehensive Cancer Cell Line Encyclopedia (CCLE) [15]. Considering 30 breast cancer cell lines and 14 targeted agents, both stratified into subtypes, we still observe a largely varying compound sensitivity rather than subtype-specific responses in the corresponding cell lines (Additional file 1: Figure S4). Thus, even when accounting for subtype-specific differences in the NCI-60, or considering a much larger cell line panel with a broader representation of breast cancer subtypes [15] (see Additional file 1: Figure S4), the previously observed tendencies regarding tissue specificity still hold.

Similarly, we investigated whether intended drug targets are expressed in the corresponding cell lines and, if so, whether the drug is active in this cell line, exploiting proteomics data of NCI-60 [16]. Again, we could not correlate target expression with drug sensitivity (Additional file 1: Figure S5). One of the few exceptions is presented by the set of EGFR modulators, Gefitinib and Erlotinib, to which cell lines are indeed more sensitive when EGFR is expressed. Yet, in the majority of instances drug activity does not depend on the presence or absence of the intended targets, which indicates that several factors, beyond target expression, determine drug sensitivity.

Although alternative models for drug screening and development are generated [9], cancer cell lines have been and will be an essential component of cancer research and drug discovery [17]. However, as we observed, relying on assays performed in a few selected cell lines may result in incorrect or misleading conclusion, and thus is unlikely to predict clinical outcomes. Cell line panels, on the other hand, may embrace the underlying complexity and variability of cancer. Yet, to fully exploit their invaluable potential, we have to move beyond ‘one marker, one cell line’ studies and incorporate the large amount of molecular (‘omics’) profiles into robust systems biology frameworks [15,16,18-20]. We believe that identifying and combining the key features that each cell line is able to reproduce, beyond tissue and subtype specificity, will bring screening panels at the forefront of a more successful drug discovery.

## Additional files

Additional file 1: Table S1.
**Summary of cancer-specific drugs assembled and mapped per cancer type.** BC – breast cancer, CRC – colorectal cancer, PC – prostate cancer. Note that some drugs could be mapped onto several NCI-60 tested compounds. **Table S3.** Classification of targeted breast cancer agents tested in the NCI-60 into distinct subtypes according to the FDA Orange Book, the NCI, ClinicalTrials.gov and the literature. B – basal/triple negative, HR – hormone receptor positive, H2 – HER2-overexpressing cancer, U – subtype indication unknown. **Table S4.** Classification of targeted breast cancer agents, screened in the CCLE, into distinct subtypes according to the FDA Orange Book, the NCI, ClinicalTrials.gov and the literature. B – basal/triple negative, HR – hormone receptor positive, H2 – HER2-overexpressing cancer, U – subtype indication unknown. **Figure S1.** Sensitivity of the breast cancer-specific drug sets considering (A) approved and (B) targeted drugs, (C) normalized drug sensitivities, as well as (D) Unique and (E) Unique* drugs. **Figure S2.** Sensitivity of the colorectal cancer-specific drug sets considering (A) approved and (B) targeted drugs, (C) normalized drug sensitivities, as well as (D) Unique and (E) Unique* drugs. **Figure S3.** Sensitivity of the prostate cancer-specific drug sets considering (A) approved and (B) targeted drugs, (C) normalized drug sensitivities, as well as the (D) Unique and (E) Unique* sets. **Figure S4.** Sensitivity of drugs indicated for hormone receptor positive, triple negative and HER2-overexpressing breast cancer within 30 breast cancer cell lines representing the different subtypes. Sensitivity is indicated in terms of the IC50. Red cells indicate high activity while green cells indicate inactivity. **Figure S5.** Contingency tables grouping target expression and discretized drug sensitivity of cell lines. Repeated instances, such as EPHA2 – Dasatinib, correspond to different NCI-60 compounds, i.e., different formulation of the same drug. Additional file 2: Table S2. Overview on the cancer drugs assembled from DrugBank, TTD and the NCI for breast, colorectal and prostate cancer, including the mapping to NCI-60 molecules and information on the drug status (approved), the agent type (targeted agents) and whether they are used to treat other diseases (Unique and Unique*).
