# Supplementary material for: Drug sensitivity in cancer cell lines is not tissue-specific
Source: Mol Cancer. 2015 Feb 15;14:40. doi: 10.1186/s12943-015-0312-6 (PMC4359490; doi:10.1186/s12943-015-0312-6)

**Drug sensitivity in cancer cell lines is not tissue-specific**

Samira Jaeger*, Miquel Duran-Frigola*, and Patrick Aloy

SUPPLEMENTARY INFORMATION

**Mapping cancer drugs to NCI-60 data**

For each cancer type we obtained a specific drug set assembled from the National Cancer Institute (http://www.cancer.gov/cancertopics/), DrugBank [[1](#_ENREF_1)] and the Therapeutic Target Database [[2](#_ENREF_2)]. These drugs were then mapped to NCI molecules using the PubChem repository (http://pubchem.ncbi.nlm.nih.gov), CAS number and coincidence of 2D structures (Table S1 and S2). For further analysis, we distinguished between approved and experimental agents as well as cytotoxic and targeted agents (Table S2). Cytotoxic agents comprise all drugs that interfere directly with the DNA and processes related to transcription, such as folic acid antagonists, antimetabolites and microtubule modulators.

In total, we analyzed 75 cancer drugs, 50 of which are used to treat only one of the three cancer types. There are, however, 25 drugs with overlapping therapeutic indications (i.e. 9 cytotoxic agents). To ensure that our results are not skewed by drugs effective against several cancer types, we defined two additional data sets. The first one, which we named “Unique”, comprises drugs uniquely associated with one cancer type: 19, 11 and 20 drugs for breast, colorectal and prostate cancer, respectively. We further refined these sets to include agents exclusively indicated for either breast, colorectal or prostate cancer (Unique*). The highly restricted sets comprised 10, 4 and 7 compounds for breast, colorectal and prostate cancer, respectively (Table S2).

To correct for pan-activity of some compounds we normalized the drug sensitivity across cell lines by subtracting the mean GI50 and dividing by the standard deviation. All the results are reported in Figures S1, S2 and S3.

**Comparing drug sensitivities among cancer cell lines**

To assess the tissue specificity of a cancer drug set, we compared the drug sensitivities of each cancer-specific cell line against the sensitivities of cell lines not reflecting the cancer type, using the Wilcoxon test. In breast cancer, only the MCF-7 line exhibited a significantly higher sensitivity with respect to 11 and 1 other cell lines when considering a threshold α of 0.05 and 0.01, respectively. A similar case was also observed for colorectal and prostate cancer, with only HCT-116 (CRC) and DU-145 (PC), being significantly more sensitive than 7 and 2 other cell lines, for α of 0.05 and 0.01, respectively. However, when we applied a Bonferroni correction for multiple testing the previously detected differences vanished for all cell lines, indicating that the modest differences between the cancer cell lines are not globally significant. Bonferroni corrected thresholds were α = 0.00092, α = 0.00096, α = 0.00087 for breast, colorectal and prostate cancer. If we consider the more specific, but smaller, drug sets, Unique and Unique*, no significant differences can be identified with respect to the sensitivity for any set or cancer type.

**Sensitivity of breast cancer drugs in the Cancer Cell Line Encyclopedia (CCLE)**

The NCI-60 only involves five breast cancer cell lines, which weakly reflect the distinct subtypes. Thus, we performed a similar analysis considering the more comprehensive Cancer Cell Line Encyclopedia (CCLE) [[3](#_ENREF_3)]. The CCLE screened 24 compounds across 504 cancer cell lines of which 30 are breast cancer cell lines (Table S4). 21 compounds represent targeted agents such as kinase inhibitors, which range from preclinical to launched stages, only one of them approved for breast cancer, namely lapatinib. For compounds indicated for breast cancer treatment, in terms of clinical trials or other preclinical studies, we carried out the same analysis as described for the NCI-60. First, we classified the 30 breast cancer lines according to their characteristics into HR-positive (9), triple negative/basal (13) and HER2-overexpressiong (5) subtypes (3 cell lines could not be classified). Next, we divided the 14 targeted breast cancer agents according to available indications into subtypes. Note that the majority of agents does not correspond to the typical breast cancer agents, such as PARP inhibitors, selective estrogen modulators, aromatase inhibitors or mTOR inhibitors. Hence, the classification of these agents is more challenging (four of them could not be classified). Given the drug and cell line sets, we assessed whether we can observe subtype-specific activities for the corresponding compounds. Figure S4 indicates that the sensitivity of compounds varies largely across breast cancer cells. Furthermore, even when considering a broader collection of subtype-specific cell lines, we do not observe subtype-specific responses in the corresponding cell lines.

**Drug sensitivity with respect to protein expression**

Given the weak correlation between tissue type sensitivity and drug indication, we investigated whether intended drug targets are expressed in the corresponding cell lines, and, if so, whether the drug is active in this cell line. For this, we considered the large-scale proteomics data generated by Moghaddas et al [[4](#_ENREF_4)].

To enable a more systematic consideration of drug sensitivity with respect to target expression, we discretized drug activities into three categories, i.e., resistant, intermediate and sensitive. To do so, we used the waterfall method first described in the CCLE study [[3](#_ENREF_3)] and applied more recently by Haibe-Kains et al. [[5](#_ENREF_5)], which considers the general specificity of a compound when assessing the sensitivity of a cell line. We applied the following strategy: First, we extracted drug sensitivity measurements, i.e., GI50, from NCI-60. For each compound we then sorted the cell lines according to increasing GI50s to generate a waterfall distribution. For nonlinear waterfall distribution with a Pearson correlation coefficient ≤ 0.95, we estimated the major inflection point of the GI50 curve as the point of the curve with the maximal distance to a line between the start and the endpoints. Otherwise, we used the median GI50 as inflection point. Cell lines within a four-fold GI50 difference around this inflection point are classified as intermediate, cell lines with a lower GI50 are defined to be sensitive while those with a larger GI50 are classified as resistant.

Similarly, we classified a protein as expressed if it was detected in the large-scale proteomics profiling of the NCI60 [[4](#_ENREF_4)], and not expressed otherwise. Proteomics data covered a total of 8,113 proteins.

Figure S5 groups the discretized activity of several drugs according to the presence or absence of their intended targets. As it can be seen, in general, cell lines expressing the intended target are not more sensitive to the targeted therapy.

REFERENCES

1. Knox C, Law V, Jewison T, Liu P, Ly S, Frolkis A, Pon A, Banco K, Mak C, Neveu V *et al*: **DrugBank 3.0: a comprehensive resource for 'omics' research on drugs**. *Nucleic Acids Res* 2011, **39**(Database issue):D1035-1041.

2. Qin C, Zhang C, Zhu F, Xu F, Chen SY, Zhang P, Li YH, Yang SY, Wei YQ, Tao L *et al*: **Therapeutic target database update 2014: a resource for targeted therapeutics**. *Nucleic Acids Res* 2014, **42**(Database issue):D1118-1123.

3. Barretina J, Caponigro G, Stransky N, Venkatesan K, Margolin AA, Kim S, Wilson CJ, Lehar J, Kryukov GV, Sonkin D *et al*: **The Cancer Cell Line Encyclopedia enables predictive modelling of anticancer drug sensitivity**. *Nature* 2012, **483**(7391):603-607.

4. Moghaddas Gholami A, Hahne H, Wu Z, Auer FJ, Meng C, Wilhelm M, Kuster B: **Global proteome analysis of the NCI-60 cell line panel**. *Cell Rep* 2013, **4**(3):609-620.

5. Haibe-Kains B, El-Hachem N, Birkbak NJ, Jin AC, Beck AH, Aerts HJ, Quackenbush J: **Inconsistency in large pharmacogenomic studies**. *Nature* 2013, **504**(7480):389-393.

Table S1: Summary of cancer-specific drugs assembled and mapped per cancer type. BC – breast cancer, CRC – colorectal cancer, PC – prostate cancer. Note that some drugs could be mapped onto several NCI-60 tested compounds).

| **Cancer type** | **Drugs** | **Drugs mapped to NCI60** | **∑ NCI60**  **compounds** | **Approved agents** | **Targeted agents** |
| --- | --- | --- | --- | --- | --- |
| BC | 66 | 41 | 45 | 28 | 23 |
| CRC | 72 | 24 | 24 | 7 | 14 |
| PC | 91 | 38 | 42 | 9 | 27 |

Table S2: Overview on the cancer drugs assembled from DrugBank, TTD and the NCI for breast, colorectal and prostate cancer, including the mapping to NCI-60 molecules and information on the drug status (approved), the agent type (targeted agents) and whether they are used to treat other diseases (Unique and Unique*).

(The table is included as a separate xlsx document)

Table S3: Classification of targeted breast cancer agents tested in the NCI-60 into distinct subtypes according to the FDA Orange Book, the NCI, ClinicalTrial.gov and the literature. B – basal/triple negative, HR – hormone receptor positive, H2 – HER2-overexpressing cancer, U – subtype indication unknown.

| **Compound** | **NCI-60 ID** | **DrugBank or TTD ID** | **Subtype** | **Reference**  **(PMID or Clinical trial ID)** |
| --- | --- | --- | --- | --- |
| ABT-888 | 737664 | DNC008718 | B | NCT01104259, NCT01149083 |
| Olaparib | 747856 | DCL000583 | B | NCT02000622, NCT02032823 |
| Tanespimycin | 330507 | DCL000242 | H2 | 21558407, 23386910, NCT00817362, NCT00494234 |
| Gefitinib | 715055,  759856 | DB00317 | B | 21965336 |
| Erlotinib | 718781 | DB00530 | B | 21965336 |
| Drostanolone | 12198 | DB00858 | HR | FDA-approved |
| Testolactone | 23759 | DB00894 | HR | FDA-approved |
| Tamoxifen | 180973,  757345 | DB00675 | HR | FDA-approved |
| Aminoglutethimide | 330915 | DB00357 | HR | FDA-approved |
| Midostaurin | 656576 | DCL000165 | HR | 21965336 |
| PD-332991 | 705701 | DCL000365 | HR | NCT01942135, NCT01740427, NCT01864746, NCT01684215 |
| Exemestane | 713563 | DB00990 | HR | FDA-approved |
| Fulvestrant | 719276 | DB00947 | HR | FDA-approved |
| Anastrozole | 719344 | DB01217 | HR | FDA-approved |
| Letrozole | 719345 | DB01006 | HR | FDA-approved |
| Raloxifene | 747974 | DB00481 | HR | FDA-approved |
| Vandetanib | 760766 | DB05294 | HR | 24526731 |
| Sorafenib | 747971 | DB00398 | B, HR | NCT01234337 |
| Lapatinib | 745750 | DB01259 | HR, H2 | FDA-approved |
| Temsirolimus | 683864 | DB06287 | HR, H2 | 23386910, 21965336 |
| Everolimus | 733504 | DB01590 | HR, H2 | 23386910, 21965336 |
| Figitumumab | 677083 | DCL000527 | HR | 23386910 |
| Exherin | 729477 | DCL000110 | U |  |

Table S4: Classification of targeted breast cancer agents, screened in the CCLE, into distinct subtypes according to the FDA Orange Book, the NCI, ClinicalTrial.gov and the literature. B – basal/triple negative, HR – hormone receptor positive, H2 – HER2-overexpressing cancer, U – subtype indication unknown.

| **Compound** | **Subtype** | **Reference**  **(PMID or Clinical trial Id)** |
| --- | --- | --- |
| Erlotinib | B | 21965336 |
| AEW541 | HR, H2 | 23117852 |
| Tanespimycin | H2 | 23386910 |
| Vandetanib | HR | 24526731 |
| Panobinostat | B | 22613095, 24366407 |
| Lapatinib | HR, H2 | FDA-approved |
| Sorafenib | B, HR | NCT01234337 |
| PD-332991 | HR | NCT01942135, NCT01740427, NCT01864746, NCT01684215, NCT02040857 |
| AZD0530 | HR | ARISTACAT trial |
| TKI258 | HR | NCT01528345 |
| Nutlin-3 | U |  |
| PHA-665752 | U |  |
| PD-325901 | U |  |
| AZD6244 | U |  |

Figure S1: Sensitivity of the breast cancer-specific drug sets considering (A) approved and (B) targeted drugs, (C) normalized drug sensitivities, as well as (D) Unique and (E) Unique* drugs.


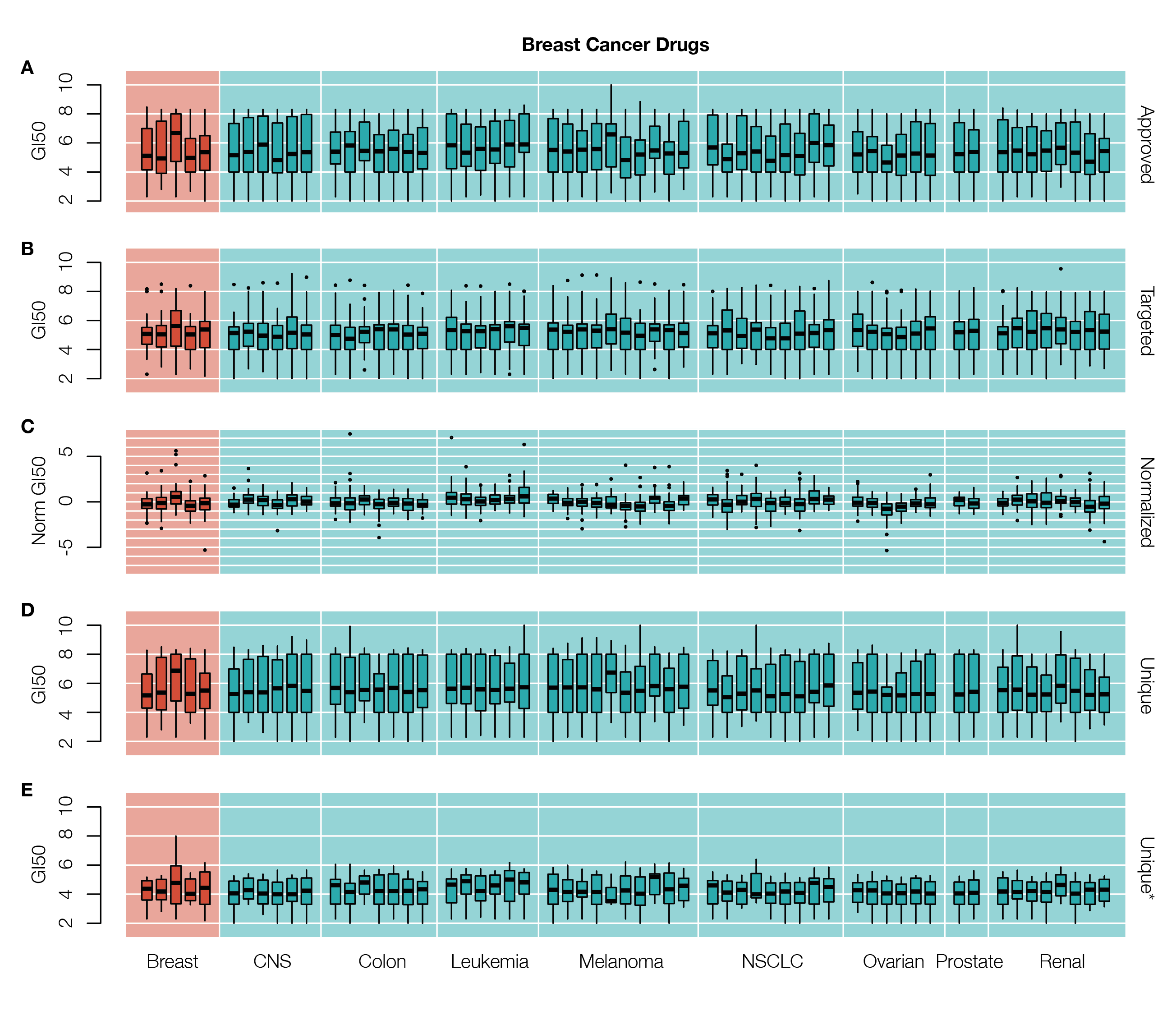


Figure S2: Sensitivity of the colorectal cancer-specific drug sets considering (A) approved and (B) targeted drugs, (C) normalized drug sensitivities, as well as (D) Unique and (E) Unique* drugs.


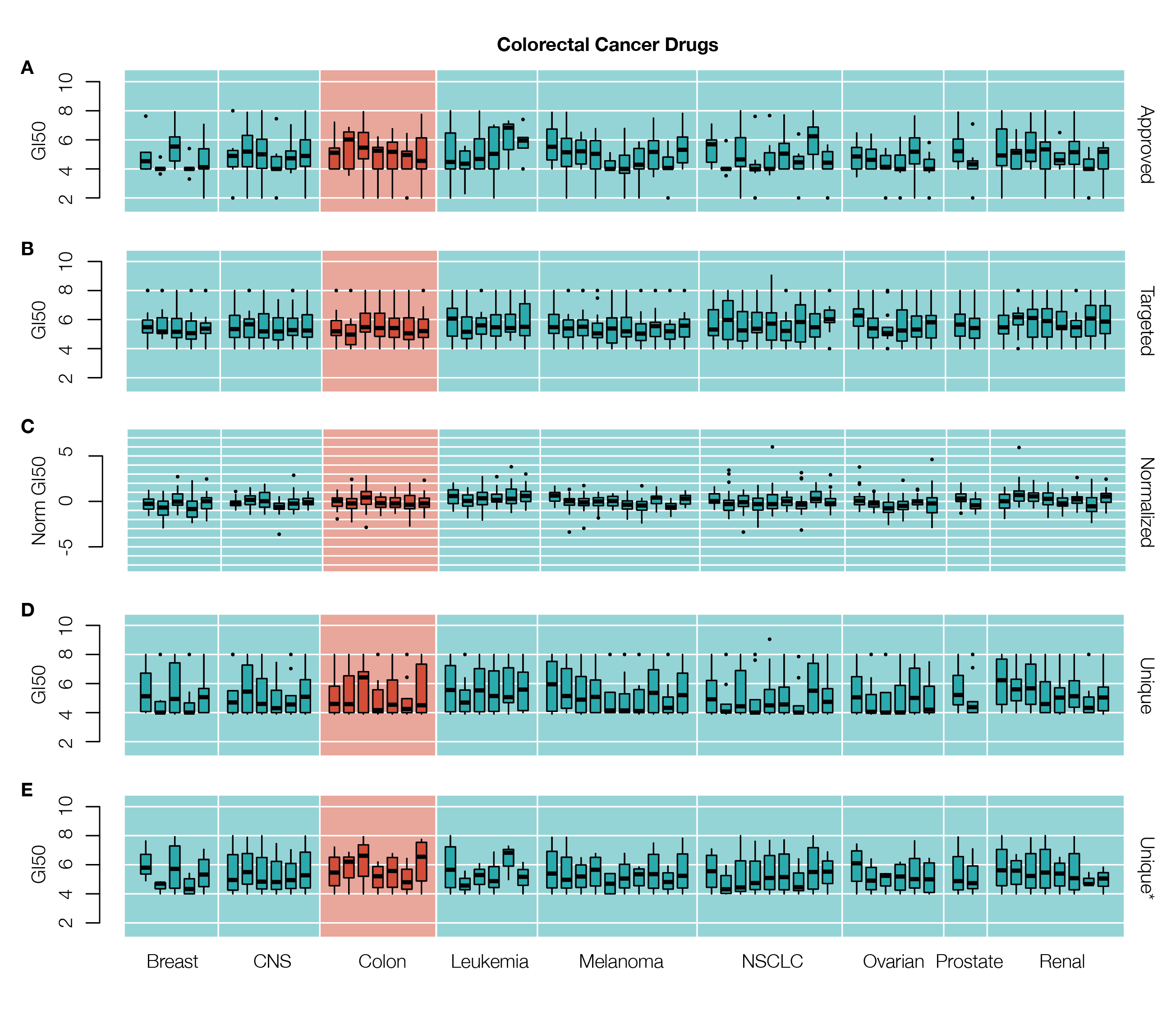


Figure S3: Sensitivity of the prostate cancer-specific drug sets considering (A) approved and (B) targeted drugs, (C) normalized drug sensitivities, as well as the (D) Unique and (E) Unique* sets.


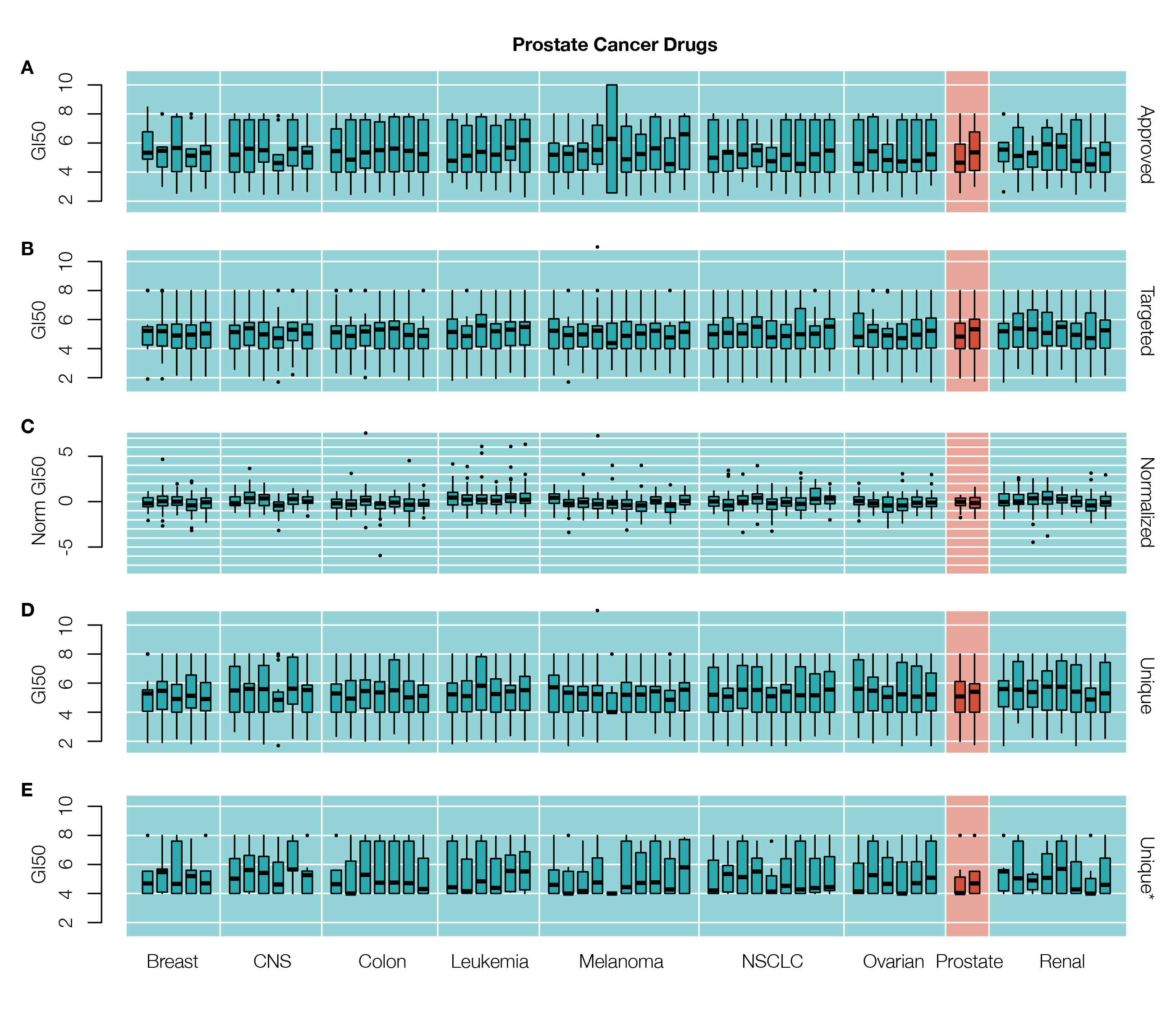


Figure S4: Sensitivity of drugs indicated for hormone receptor positive, triple negative and HER2-overexpressing breast cancer within 30 breast cancer cell lines representing the different subtypes. Sensitivity is indicated in terms of the IC50. Red cells indicate high activity while green cells indicate inactivity.


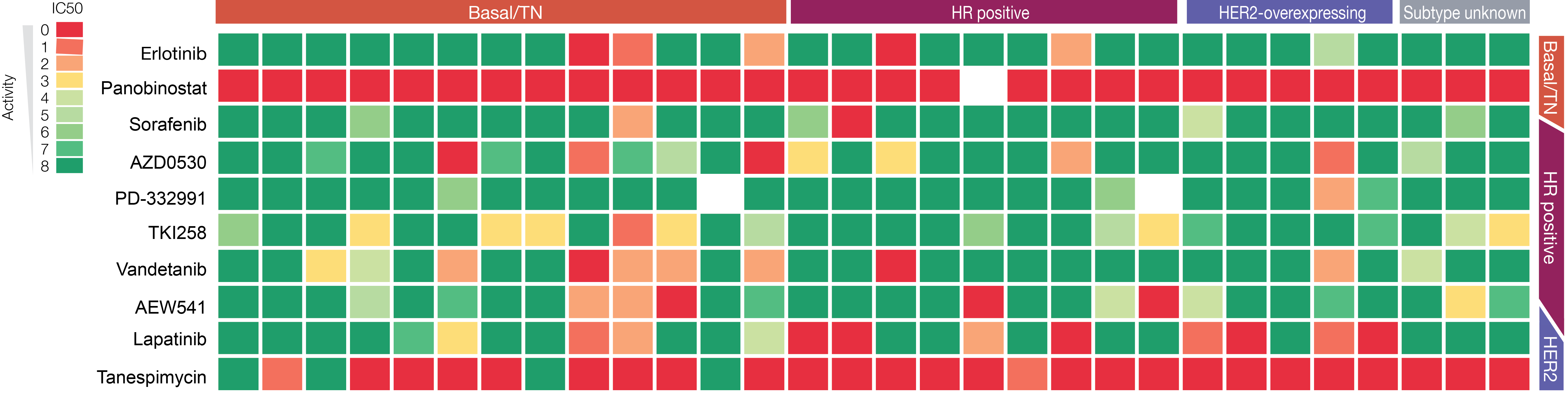


Figure S5: Contingency tables grouping target expression and discretized drug sensitivity of cell lines. Repeated instances, such as EPHA2 – Dasatinib, correspond to different NCI-60 compounds, i.e., different formulation of the same drug.


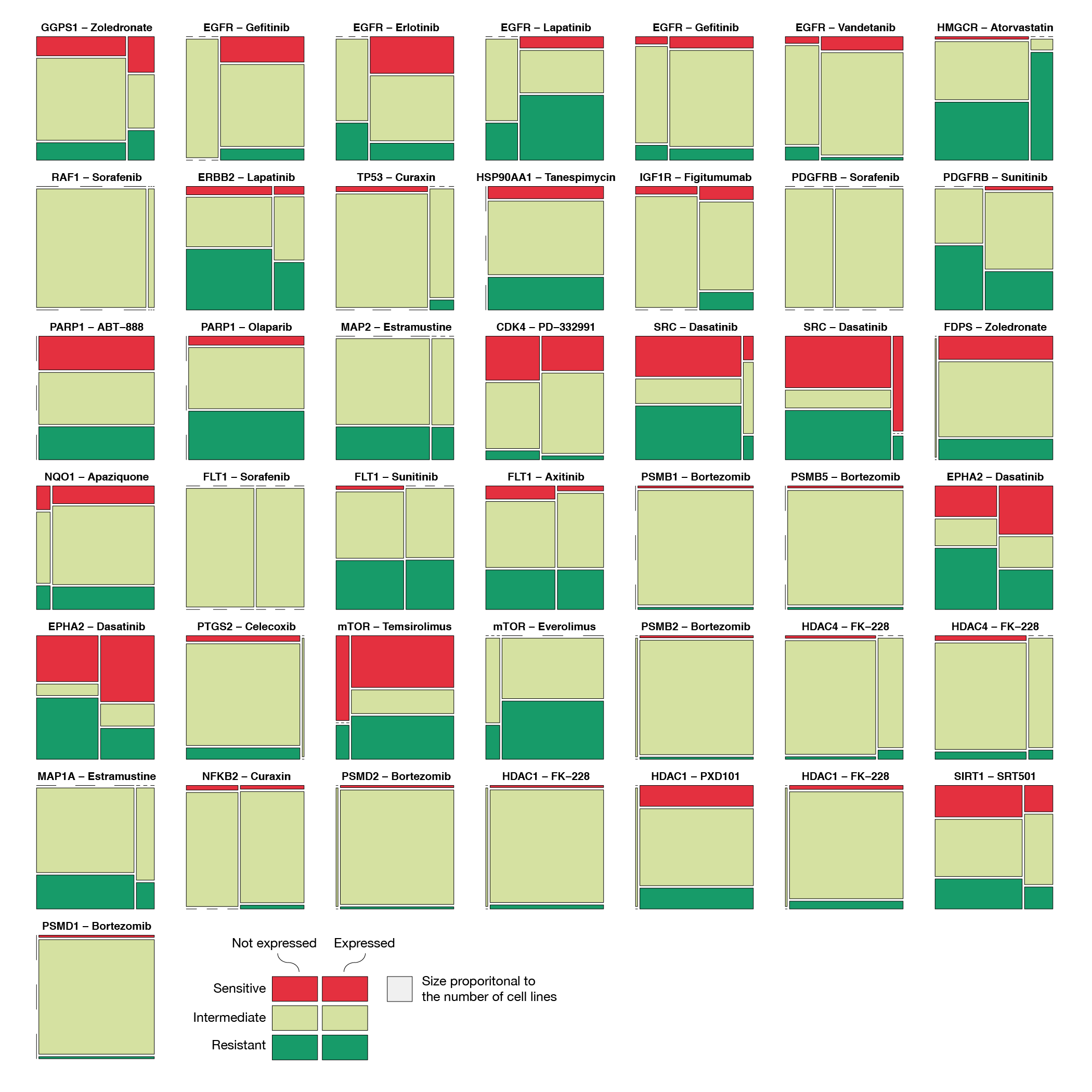

Supplement: Additional file 1: Table S1. — Summary of cancer-specific drugs assembled and mapped per cancer type. BC – breast cancer, CRC – colorectal cancer, PC – prostate cancer. Note that some drugs could be mapped onto several NCI-60 tested compounds. Table S3. Classification of targeted breast cancer agents tested in the NCI-60 into distinct subtypes according to the FDA Orange Book, the NCI, ClinicalTrials.gov and the literature. B – basal/triple negative, HR – hormone receptor positive, H2 – HER2-overexpressing cancer, U – subtype indication unknown. Table S4. Classification of targeted breast cancer agents, screened in the CCLE, into distinct subtypes according to the FDA Orange Book, the NCI, ClinicalTrials.gov and the literature. B – basal/triple negative, HR – hormone receptor positive, H2 – HER2-overexpressing cancer, U – subtype indication unknown. Figure S1. Sensitivity of the breast cancer-specific drug sets considering (A) approved and (B) targeted drugs, (C) normalized drug sensitivities, as well as (D) Unique and (E) Unique* drugs. Figure S2. Sensitivity of the colorectal cancer-specific drug sets considering (A) approved and (B) targeted drugs, (C) normalized drug sensitivities, as well as (D) Unique and (E) Unique* drugs. Figure S3. Sensitivity of the prostate cancer-specific drug sets considering (A) approved and (B) targeted drugs, (C) normalized drug sensitivities, as well as the (D) Unique and (E) Unique* sets. Figure S4. Sensitivity of drugs indicated for hormone receptor positive, triple negative and HER2-overexpressing breast cancer within 30 breast cancer cell lines representing the different subtypes. Sensitivity is indicated in terms of the IC50. Red cells indicate high activity while green cells indicate inactivity. Figure S5. Contingency tables grouping target expression and discretized drug sensitivity of cell lines. Repeated instances, such as EPHA2 – Dasatinib, correspond to different NCI-60 compounds, i.e., different formulation of the same drug. [file 12943_2015_312_MOESM1_ESM.docx]
